# Extensive Disease of the Gums and Alveoli from a Decayed Tooth

**Published:** 1850-01

**Authors:** Saml. Griffith

**Affiliations:** Louisville, Ky.


*Extract from the Western Journal of Medicine and Surgery.*

### Art. 111. EXTENSIVE DISEASE OF THE GUMS AND ALVEOLI FROM A DECAYED TOOTH.

BY SAML. GRIFFITH, D. D. S.—*Louisville, Ky.*

Mr. B., of Barnwell C. H., Barnwell District, South Carolina, consulted me, in June, 1831, on account of a disease of the alveolar processes of the lower jaw, of five years standing. The patient was in a most wretched condition. He carried the head on the left shoulder, the right side of the neck being so much swollen as to be on a level with the point of the right shoulder.

On carrying the finger into the mouth, which required considerable force, I found the teeth covered with a thick coating of brown tartar, the gums in a high state of inflammation, spongy, and bleeding on the slightest touch. The only tooth that had any appearance of decay, was the first molar on the left side, and it was in this that the disease had commenced. From this point to the dens sapientia of the right side, purulent matter issued freely from around the necks of all the teeth.

Sounding the last named tooth, produced a sensation in the patient like that of an electric shock. Pieces of the alveolar arch had exfoliated, and beneath the right wisdom tooth was a portion of the jaw bone partially detached; it was an inch and a quarter long, and about a quarter of an inch thick, partly porous and cellular, partly solid. The immense swelling of the neck did not contain any matter, but seemed to depend mainly upon imflammation of the glands and cellular tissue.

The disease, as I learned from the patient and his physician was of five years duration, having resisted all the efforts of the best medical talent in the country. An immense amount of matter had been discharged during this time, and from having been a stout, athletic man, Mr. B. was reduced to an extreme degree of feebleness.

June 15th. I began the treatment by extracting the wisdom tooth of the right side,* and removing as much of the tartar as could be reached conveniently. This operation was extremely painful, and had to be desisted from very soon. I prescribed a mild astringent wash for the mouth, to be used frequently, and gentle cathartics to keep the bowels open.

June 23d. I saw Mr. B. again. The swelling of the neck had very much diminished, the head was more erect, the mouth could be opened more easily, and there was less inflammation of the gums. I cleansed the teeth still further, and removed three pieces of bone from the alveolar arch. Continued the wash for the mouth, a little stronger than at first, and directed the cathartics as before.

July 2d. Mr. B. called on me again. Swelling of the neck and inflammation of the mouth quite gone, and the head nearly erect. This time I succeeded in removing all the tartar. I took away, also, three more pieces of the alveolar arch, and large pieces of the bone described above. Medicines continued as before.

July 12th. Saw Mr. B., he was perfectly restored, head

* This appeared perfectly healthy after extraction. Upon splitting it open, however, the cavity was filled with purulent matter, and the nerve and lining membrane were quite destroyed.

erect, and mouth healthy. I saw him frequently afterwards, and he informed me he had never suffered any since his mouth first healed.

July, 1844.

